# 2,2′-[(1*S*,2*S*)-1,2-Bis(2-hy­droxy­phen­yl)ethane-1,2-di­yl]bis(isoindoline-1,3-dione) ethanol monosolvate hemihydrate

**DOI:** 10.1107/S1600536813003978

**Published:** 2013-02-20

**Authors:** Jik Chin, Dongsoo Koh, Alan J. Lough

**Affiliations:** a Department of Chemistry, University of Toronto, Toronto, Ontario, Canada M5S 3H6; b Department of Applied Chemistry, Dongduk Women’s University, Seoul 136-714, Republic of Korea

## Abstract

In the title compound, C_30_H_20_N_2_O_6_·C_2_H_6_O·0.5H_2_O, the solvent water mol­ecule lies on a twofold rotation axis. The dihedral angle between the essentially planar isoindole ring systems [maximum deviations = 0.028 (1) and 0.022 (1) Å] is 47.12 (5)°. The dihedral angle between the benzene rings is 81.32 (7)°. In the crystal, the components are linked into a three-dimensional network *via* O—H⋯O hydrogen bonds.

## Related literature

For the use of chiral bis­phenolic ligands in stereoselective catalysis, see: Noyori *et al.* (1984 ▶); Takaya *et al.* (1989 ▶); Liu & Ding (2005 ▶); Xu *et al.* (2011 ▶); Yamaguchi *et al.* (2009 ▶); Van den Berg *et al.* (2002 ▶); So *et al.* (2012 ▶); Kim, Nguyen *et al.* (2008 ▶); Kim, So *et al.* (2008 ▶); For related structures, see: Li *et al.* (2011 ▶); Liu *et al.* (2011 ▶). For analysis of the absolute configration, see: Hooft *et al.* (2008 ▶).
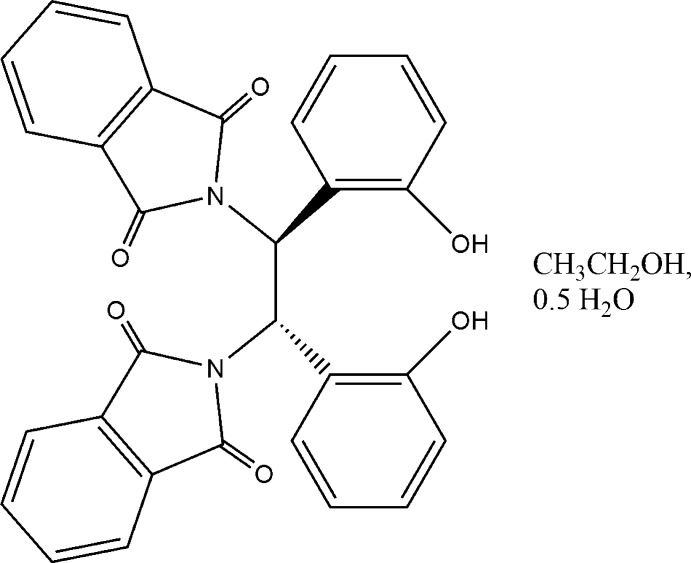


## Experimental

### 

#### Crystal data

C_30_H_20_N_2_O_6_·C_2_H_6_O·0.5H_2_O
*M* *_r_* = 559.56
Tetragonal, 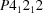

*a* = 10.6848 (3) Å
*c* = 47.9935 (17) Å
*V* = 5479.2 (3) Å^3^
*Z* = 8
Cu *K*α radiation
μ = 0.81 mm^−1^
*T* = 147 K
0.29 × 0.18 × 0.18 mm

#### Data collection

Bruker Kappa APEX DUO CCD diffractometer
Absorption correction: multi-scan (*SADABS*; Bruker, 2007 ▶) *T*_min_ = 0.696, *T*_max_ = 0.753
35250 measured reflections
4786 independent reflections
4756 reflections with *I* > 2σ(*I*)
*R*_int_ = 0.035

#### Refinement

*R*[*F* ^2^ > 2σ(*F* ^2^)] = 0.029
*wR*(*F* ^2^) = 0.075
*S* = 1.12
4786 reflections
391 parameters
H atoms treated by a mixture of independent and constrained refinement
Δρ_max_ = 0.26 e Å^−3^
Δρ_min_ = −0.18 e Å^−3^
Absolute structure: Flack (1983 ▶), 1895 Friedel pairs
Flack parameter: 0.03 (13)

### 

Data collection: *APEX2* (Bruker, 2007 ▶); cell refinement: *SAINT* (Bruker, 2007 ▶); data reduction: *SAINT*; program(s) used to solve structure: *SHELXS97* (Sheldrick, 2008 ▶); program(s) used to refine structure: *SHELXL97* (Sheldrick, 2008 ▶); molecular graphics: *PLATON* (Spek, 2009 ▶); software used to prepare material for publication: *SHELXTL* (Sheldrick, 2008 ▶).

## Supplementary Material

Crystal structure: contains datablock(s) I, global. DOI: 10.1107/S1600536813003978/aa2082sup1.cif

Structure factors: contains datablock(s) I. DOI: 10.1107/S1600536813003978/aa2082Isup2.hkl

Supplementary material file. DOI: 10.1107/S1600536813003978/aa2082Isup3.cml

Additional supplementary materials:  crystallographic information; 3D view; checkCIF report

## Figures and Tables

**Table 1:** Hydrogen-bond geometry (Å, °)

*D*—H⋯*A*	*D*—H	H⋯*A*	*D*⋯*A*	*D*—H⋯*A*
O1—H1*O*⋯O1*W*	0.86 (2)	1.89 (2)	2.7263 (13)	165 (2)
O2—H2*O*⋯O5^i^	0.88 (2)	1.95 (2)	2.8238 (15)	177 (2)
O1*S*—H1*SO*⋯O4	1.06 (4)	1.76 (4)	2.7905 (17)	161 (3)
O1*W*—H1*W*⋯O1*S* ^ii^	1.06 (3)	1.65 (3)	2.6927 (16)	169 (3)

Symmetry codes: (i) 
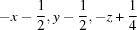
; (ii) 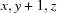.

**Fig. 1.**
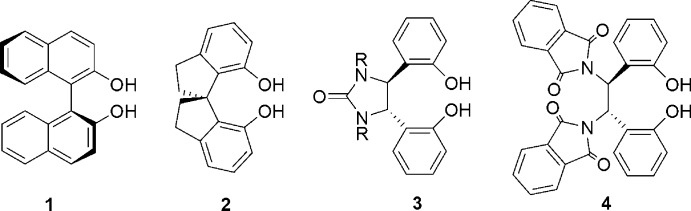
Chiral bisphenolic ligands used for stereoselective catalysis.

**Fig. 2.**
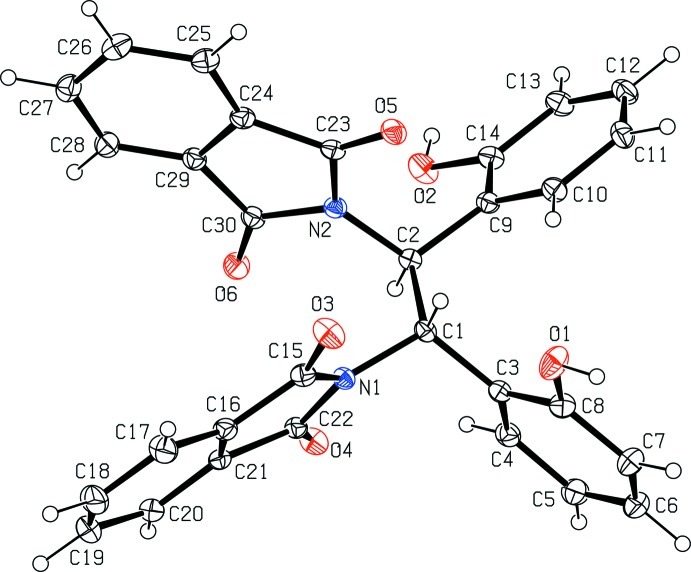
The molecular structure of the title compound with 30% probability ellipsoids. The ethanol solvent molecule and hemihydrate are not shown.

**Fig. 3.**
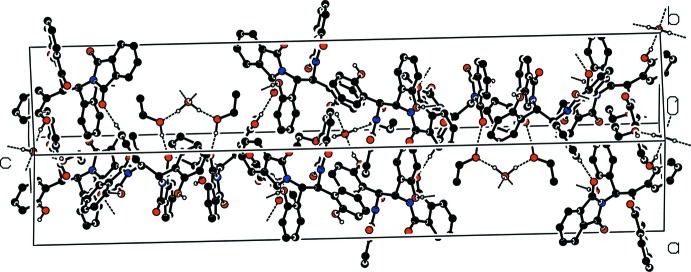
Part of the crystal structure with hydrogen bonds shown as dashed lines.

## supplementary crystallographic information

### Comment

Binol (1, see Fig. 1) is a privileged structure for developing a wide variety of stereoselective catalysts (Noyori *et al.*, 1984; Takaya *et al.*, 1989). More recently, other chiral bisphenolic compounds (2, 3) have also gained popularity (Liu & Ding, 2005; Xu *et al.*, 2011). The phenolic O atoms in these compounds are useful for chelating to metals (Yamaguchi *et al.*, 2009) or for forming monophos ligands (Van den Berg *et al.*, 2002). We have shown that bis-(2-hydroxyphenyl)-1,2-diaminoethane (*hpen*) is a highly useful chiral diamine for making many other chiral diamines by diaza-Cope rearrangement (So *et al.*, 2012; Kim, Nguyen *et al.*, 2008; Kim, So *et al.*, 2008). Here we report a structure of a chiral bisphenolic compound (4) derived from *hpen* by simple protection of the amino groups as phthalimides (Li *et al.*, 2011; Liu *et al.*, 2011). As anticipated from molecular mechanics computation, the structure reveals that the two phenol groups in 4 are in a *gauche* arrangement.

The molecular structure of the title compound (4) is shown in Fig. 2. The solvent water molecule lies on a twofold rotation axis. The dihedral angle between the essentially planar isoindole ring systems [N1/C15—C22 and N2/C23—C30, with maximum deviations of 0.028 (1) for C15 and 0.022 (1) Å for C24) is 47.12 (5)°. The dihedral angle between the two benzene rings [C3—C8 and C9—C14 is 81.32 (7)°]. In the crystal, the components of the structure are linked into a three-dimensional network *via* O—H···O hydrogen bonds (Fig. 3).

### Experimental

To a suspension of phthalic anhydride (1.48 g, 10 mmol) in 10 ml of acetic acid was added 1,2-bis(2-hydroxyphenyl)-1,2-diaminoethane (1.22 g, 5 mmol). The reaction mixture was heated at 383 K for 16 h and cooled down to room temperature to give the product as off white precipitate. After filtration, recrystallization of the title compound in ethanol gave X-ray quality crystals in 58% yield (1.47 g).

### Refinement

H atoms bonded to C atoms were included in calculated positions with C—H = 0.95–0.99 Å and included in the refinement in a riding-motion approximation with *U*_iso_(H) = 1.2*U*_eq_(C) or 1.5*U*_eq_(C_methyl_). H atoms bonded to O atoms were refined independently with isotropic displacement parameters. Analysis of the absolute configration was also performed using likelihood methods (Hooft *et al.*, 2008) as implemented in *PLATON* (Spek, 2009). The resulting value for the Hooft parameter is *y* = 0.04 (3).

### Crystal data

**Table:**

C_30_H_20_N_2_O_6_·C_2_H_6_O·0.5H_2_O	*D*_x_ = 1.357 Mg m^−^^3^
*M**_r_* = 559.56	Cu *K*α radiation, λ = 1.54178 Å
Tetragonal, *P*4_1_2_1_2	Cell parameters from 9692 reflections
Hall symbol: P 4abw 2nw	θ = 3.7–66.5°
*a* = 10.6848 (3) Å	µ = 0.81 mm^−^^1^
*c* = 47.9935 (17) Å	*T* = 147 K
*V* = 5479.2 (3) Å^3^	Needle, pale yellow
*Z* = 8	0.29 × 0.18 × 0.18 mm
*F*(000) = 2344	

### Data collection

**Table:**

Bruker Kappa APEX DUO CCD diffractometer	4786 independent reflections
Radiation source: Bruker ImuS	4756 reflections with *I* > 2σ(*I*)
Multi-layer optics monochromator	*R*_int_ = 0.035
φ and ω scans	θ_max_ = 66.6°, θ_min_ = 3.7°
Absorption correction: multi-scan (*SADABS*; Bruker, 2007)	*h* = −11→12
*T*_min_ = 0.696, *T*_max_ = 0.753	*k* = −12→12
35250 measured reflections	*l* = −53→56

### Refinement

**Table:**

Refinement on *F*^2^	Secondary atom site location: difference Fourier map
Least-squares matrix: full	Hydrogen site location: inferred from neighbouring sites
*R*[*F*^2^ > 2σ(*F*^2^)] = 0.029	H atoms treated by a mixture of independent and constrained refinement
*wR*(*F*^2^) = 0.075	*w* = 1/[σ^2^(*F*_o_^2^) + (0.0389*P*)^2^ + 1.2163*P*] where *P* = (*F*_o_^2^ + 2*F*_c_^2^)/3
*S* = 1.12	(Δ/σ)_max_ = 0.001
4786 reflections	Δρ_max_ = 0.26 e Å^−^^3^
391 parameters	Δρ_min_ = −0.18 e Å^−^^3^
0 restraints	Absolute structure: Flack (1983), 1895 Friedel pairs
Primary atom site location: structure-invariant direct methods	Flack parameter: 0.03 (13)

### Special details

**Table:**

Geometry. All s.u.'s (except the s.u. in the dihedral angle between two l.s. planes) are estimated using the full covariance matrix. The cell s.u.'s are taken into account individually in the estimation of s.u.'s in distances, angles and torsion angles; correlations between s.u.'s in cell parameters are only used when they are defined by crystal symmetry. An approximate (isotropic) treatment of cell s.u.'s is used for estimating s.u.'s involving l.s. planes.
Refinement. Refinement of *F*^2^ against ALL reflections. The weighted *R*-factor *wR* and goodness of fit *S* are based on *F*^2^, conventional *R*-factors *R* are based on *F*, with *F* set to zero for negative *F*^2^. The threshold expression of *F*^2^ > σ(*F*^2^) is used only for calculating *R*-factors(gt) *etc*. and is not relevant to the choice of reflections for refinement. *R*-factors based on *F*^2^ are statistically about twice as large as those based on *F*, and *R*- factors based on ALL data will be even larger.

### Fractional atomic coordinates and isotropic or equivalent isotropic displacement parameters (Å^2^)

**Table:**

	*x*	*y*	*z*	*U*_iso_*/*U*_eq_	
O1	−0.12757 (12)	0.57392 (11)	0.02270 (2)	0.0380 (3)	
O2	−0.27045 (11)	0.13393 (10)	0.10646 (2)	0.0335 (3)	
O3	0.16202 (10)	0.49860 (10)	0.05562 (2)	0.0337 (3)	
O4	0.03625 (10)	0.10133 (9)	0.03340 (2)	0.0285 (2)	
O5	−0.05120 (10)	0.50131 (9)	0.11171 (2)	0.0276 (2)	
O6	0.05013 (10)	0.09166 (9)	0.09551 (2)	0.0295 (2)	
N1	0.06521 (10)	0.31129 (11)	0.04480 (2)	0.0198 (2)	
N2	−0.02832 (11)	0.29340 (11)	0.09907 (2)	0.0206 (2)	
C1	−0.06345 (12)	0.35618 (13)	0.05051 (3)	0.0202 (3)	
H1A	−0.0559	0.4460	0.0561	0.024*	
C2	−0.11822 (12)	0.28568 (13)	0.07582 (3)	0.0201 (3)	
H2A	−0.1259	0.1955	0.0705	0.024*	
C3	−0.14501 (13)	0.35352 (14)	0.02451 (3)	0.0232 (3)	
C4	−0.19143 (13)	0.24298 (15)	0.01311 (3)	0.0264 (3)	
H4A	−0.1747	0.1657	0.0222	0.032*	
C5	−0.26174 (15)	0.24368 (17)	−0.01123 (3)	0.0336 (4)	
H5A	−0.2908	0.1673	−0.0190	0.040*	
C6	−0.28897 (16)	0.35578 (18)	−0.02399 (3)	0.0371 (4)	
H6A	−0.3377	0.3567	−0.0405	0.045*	
C7	−0.24576 (17)	0.46746 (17)	−0.01282 (3)	0.0365 (4)	
H7A	−0.2656	0.5446	−0.0216	0.044*	
C8	−0.17327 (15)	0.46656 (15)	0.01131 (3)	0.0288 (3)	
C9	−0.24683 (13)	0.33031 (13)	0.08481 (3)	0.0212 (3)	
C10	−0.29333 (14)	0.44909 (14)	0.07941 (3)	0.0249 (3)	
H10A	−0.2441	0.5064	0.0690	0.030*	
C11	−0.41034 (14)	0.48579 (14)	0.08892 (3)	0.0284 (3)	
H11A	−0.4406	0.5676	0.0851	0.034*	
C12	−0.48290 (14)	0.40203 (15)	0.10405 (3)	0.0290 (3)	
H12A	−0.5633	0.4264	0.1105	0.035*	
C13	−0.43858 (14)	0.28366 (15)	0.10968 (3)	0.0285 (3)	
H13A	−0.4887	0.2263	0.1199	0.034*	
C14	−0.32052 (14)	0.24807 (14)	0.10041 (3)	0.0248 (3)	
C15	0.16844 (13)	0.39132 (13)	0.04779 (3)	0.0230 (3)	
C16	0.28018 (13)	0.31660 (14)	0.04040 (3)	0.0237 (3)	
C17	0.40449 (15)	0.35044 (16)	0.03946 (3)	0.0331 (4)	
H17A	0.4304	0.4338	0.0432	0.040*	
C18	0.49035 (15)	0.25736 (19)	0.03282 (4)	0.0397 (4)	
H18A	0.5770	0.2772	0.0322	0.048*	
C19	0.45225 (15)	0.13664 (18)	0.02712 (3)	0.0367 (4)	
H19A	0.5133	0.0752	0.0227	0.044*	
C20	0.32610 (15)	0.10254 (15)	0.02776 (3)	0.0281 (3)	
H20A	0.2997	0.0196	0.0237	0.034*	
C21	0.24159 (13)	0.19577 (14)	0.03461 (3)	0.0218 (3)	
C22	0.10333 (13)	0.19047 (13)	0.03709 (3)	0.0208 (3)	
C23	−0.00112 (13)	0.40013 (13)	0.11452 (3)	0.0210 (3)	
C24	0.10131 (13)	0.36512 (13)	0.13406 (3)	0.0229 (3)	
C25	0.16348 (15)	0.43582 (14)	0.15371 (3)	0.0273 (3)	
H25A	0.1404	0.5201	0.1573	0.033*	
C26	0.26124 (16)	0.37874 (15)	0.16801 (3)	0.0305 (3)	
H26A	0.3067	0.4254	0.1815	0.037*	
C27	0.29385 (14)	0.25485 (15)	0.16298 (3)	0.0289 (3)	
H27A	0.3613	0.2184	0.1730	0.035*	
C28	0.22891 (14)	0.18341 (15)	0.14344 (3)	0.0269 (3)	
H28A	0.2497	0.0982	0.1402	0.032*	
C29	0.13328 (13)	0.24133 (13)	0.12906 (3)	0.0229 (3)	
C30	0.05112 (13)	0.19354 (13)	0.10641 (3)	0.0225 (3)	
O1S	−0.15257 (14)	−0.07024 (14)	0.04583 (3)	0.0540 (4)	
C1S	−0.1589 (2)	−0.1315 (2)	0.07221 (4)	0.0479 (5)	
H1SA	−0.1368	−0.0721	0.0872	0.057*	
H1SB	−0.0987	−0.2020	0.0727	0.057*	
C2S	−0.2877 (2)	−0.1788 (2)	0.07646 (4)	0.0545 (5)	
H2SA	−0.2932	−0.2205	0.0946	0.082*	
H2SB	−0.3085	−0.2386	0.0617	0.082*	
H2SC	−0.3467	−0.1087	0.0759	0.082*	
O1W	−0.20559 (11)	0.79441 (11)	0.0000	0.0348 (4)	
H1O	−0.153 (2)	0.636 (2)	0.0128 (5)	0.052 (6)*	
H2O	−0.324 (2)	0.093 (2)	0.1168 (4)	0.046 (6)*	
H1SO	−0.068 (3)	−0.019 (3)	0.0430 (7)	0.111 (11)*	
H1W	−0.178 (3)	0.853 (3)	0.0165 (6)	0.095 (9)*	

### Atomic displacement parameters (Å^2^)

**Table:**

	*U*^11^	*U*^22^	*U*^33^	*U*^12^	*U*^13^	*U*^23^
O1	0.0515 (7)	0.0252 (6)	0.0372 (6)	0.0009 (5)	−0.0126 (5)	0.0079 (5)
O2	0.0345 (6)	0.0240 (5)	0.0420 (6)	0.0025 (5)	0.0136 (5)	0.0098 (5)
O3	0.0308 (6)	0.0229 (5)	0.0475 (6)	−0.0061 (5)	0.0054 (5)	−0.0099 (5)
O4	0.0265 (5)	0.0237 (5)	0.0352 (6)	−0.0050 (4)	0.0041 (4)	−0.0052 (4)
O5	0.0318 (5)	0.0214 (5)	0.0297 (5)	0.0065 (4)	0.0021 (4)	−0.0017 (4)
O6	0.0371 (6)	0.0197 (5)	0.0316 (5)	0.0062 (4)	−0.0032 (5)	−0.0038 (4)
N1	0.0175 (6)	0.0203 (6)	0.0215 (5)	−0.0006 (4)	0.0016 (4)	−0.0012 (5)
N2	0.0233 (6)	0.0192 (6)	0.0195 (5)	0.0015 (5)	0.0010 (5)	−0.0009 (4)
C1	0.0182 (6)	0.0199 (7)	0.0224 (6)	0.0005 (5)	0.0030 (5)	0.0008 (5)
C2	0.0208 (7)	0.0195 (7)	0.0201 (6)	−0.0001 (5)	0.0020 (5)	−0.0009 (5)
C3	0.0189 (6)	0.0292 (7)	0.0214 (7)	0.0014 (6)	0.0037 (5)	0.0007 (6)
C4	0.0204 (7)	0.0315 (8)	0.0271 (7)	−0.0018 (6)	0.0035 (6)	−0.0016 (6)
C5	0.0251 (8)	0.0449 (9)	0.0307 (8)	−0.0020 (7)	−0.0015 (6)	−0.0087 (7)
C6	0.0294 (8)	0.0562 (11)	0.0257 (8)	0.0048 (8)	−0.0051 (6)	−0.0040 (7)
C7	0.0395 (9)	0.0432 (9)	0.0268 (7)	0.0073 (7)	−0.0034 (7)	0.0067 (7)
C8	0.0283 (8)	0.0326 (8)	0.0254 (7)	0.0010 (6)	0.0021 (6)	0.0014 (6)
C9	0.0200 (7)	0.0232 (7)	0.0204 (6)	−0.0008 (5)	0.0018 (5)	−0.0021 (5)
C10	0.0247 (7)	0.0235 (7)	0.0265 (7)	−0.0008 (6)	0.0028 (6)	0.0004 (6)
C11	0.0255 (7)	0.0261 (7)	0.0336 (8)	0.0060 (6)	0.0006 (6)	−0.0020 (6)
C12	0.0214 (7)	0.0344 (8)	0.0312 (8)	0.0009 (6)	0.0055 (6)	−0.0051 (6)
C13	0.0257 (7)	0.0306 (8)	0.0291 (7)	−0.0030 (6)	0.0080 (6)	0.0010 (6)
C14	0.0269 (7)	0.0238 (7)	0.0238 (7)	−0.0003 (6)	0.0038 (6)	−0.0006 (6)
C15	0.0238 (7)	0.0237 (7)	0.0215 (7)	−0.0039 (6)	0.0024 (5)	0.0005 (6)
C16	0.0211 (7)	0.0297 (7)	0.0204 (6)	−0.0025 (6)	0.0014 (5)	0.0000 (6)
C17	0.0235 (8)	0.0395 (9)	0.0364 (8)	−0.0064 (6)	0.0008 (6)	−0.0073 (7)
C18	0.0204 (7)	0.0592 (11)	0.0396 (9)	−0.0008 (7)	0.0015 (7)	−0.0090 (8)
C19	0.0258 (8)	0.0504 (10)	0.0340 (8)	0.0102 (8)	0.0022 (7)	−0.0047 (7)
C20	0.0293 (8)	0.0325 (8)	0.0224 (7)	0.0061 (6)	0.0009 (6)	−0.0020 (6)
C21	0.0212 (7)	0.0278 (7)	0.0163 (6)	0.0004 (6)	0.0006 (5)	0.0005 (5)
C22	0.0216 (7)	0.0223 (7)	0.0186 (6)	0.0010 (6)	0.0021 (5)	0.0011 (5)
C23	0.0233 (7)	0.0208 (7)	0.0188 (6)	−0.0004 (5)	0.0055 (5)	−0.0006 (5)
C24	0.0263 (7)	0.0221 (7)	0.0204 (6)	0.0000 (6)	0.0043 (6)	0.0016 (6)
C25	0.0328 (8)	0.0248 (7)	0.0245 (7)	−0.0027 (6)	0.0020 (6)	0.0004 (6)
C26	0.0343 (8)	0.0327 (8)	0.0246 (7)	−0.0109 (7)	−0.0022 (6)	0.0018 (6)
C27	0.0259 (7)	0.0333 (8)	0.0275 (7)	−0.0028 (6)	−0.0004 (6)	0.0075 (6)
C28	0.0276 (8)	0.0266 (7)	0.0266 (7)	0.0024 (6)	0.0021 (6)	0.0041 (6)
C29	0.0227 (7)	0.0242 (7)	0.0218 (7)	0.0011 (6)	0.0034 (5)	0.0010 (6)
C30	0.0232 (7)	0.0221 (7)	0.0223 (6)	0.0020 (6)	0.0042 (5)	0.0011 (6)
O1S	0.0582 (9)	0.0541 (8)	0.0497 (8)	−0.0259 (7)	−0.0012 (7)	0.0048 (7)
C1S	0.0512 (11)	0.0473 (11)	0.0453 (10)	−0.0082 (9)	−0.0092 (9)	0.0008 (8)
C2S	0.0588 (13)	0.0618 (13)	0.0430 (10)	−0.0186 (10)	−0.0028 (9)	0.0056 (9)
O1W	0.0324 (5)	0.0324 (5)	0.0394 (9)	−0.0010 (7)	−0.0075 (5)	0.0075 (5)

### Geometric parameters (Å, º)

**Table:**

O1—C8	1.361 (2)	C12—H12A	0.9500
O1—H1O	0.86 (2)	C13—C14	1.391 (2)
O2—C14	1.3630 (19)	C13—H13A	0.9500
O2—H2O	0.88 (2)	C15—C16	1.479 (2)
O3—C15	1.2082 (18)	C16—C17	1.377 (2)
O4—C22	1.2050 (18)	C16—C21	1.383 (2)
O5—C23	1.2137 (17)	C17—C18	1.390 (2)
O6—C30	1.2078 (18)	C17—H17A	0.9500
N1—C15	1.4030 (18)	C18—C19	1.380 (3)
N1—C22	1.4033 (18)	C18—H18A	0.9500
N1—C1	1.4815 (17)	C19—C20	1.397 (2)
N2—C23	1.3909 (18)	C19—H19A	0.9500
N2—C30	1.4081 (18)	C20—C21	1.384 (2)
N2—C2	1.4749 (18)	C20—H20A	0.9500
C1—C3	1.5222 (19)	C21—C22	1.4832 (19)
C1—C2	1.5444 (18)	C23—C24	1.489 (2)
C1—H1A	1.0000	C24—C25	1.379 (2)
C2—C9	1.5173 (19)	C24—C29	1.387 (2)
C2—H2A	1.0000	C25—C26	1.391 (2)
C3—C4	1.393 (2)	C25—H25A	0.9500
C3—C8	1.397 (2)	C26—C27	1.390 (2)
C4—C5	1.389 (2)	C26—H26A	0.9500
C4—H4A	0.9500	C27—C28	1.394 (2)
C5—C6	1.376 (3)	C27—H27A	0.9500
C5—H5A	0.9500	C28—C29	1.380 (2)
C6—C7	1.387 (3)	C28—H28A	0.9500
C6—H6A	0.9500	C29—C30	1.488 (2)
C7—C8	1.393 (2)	O1S—C1S	1.427 (2)
C7—H7A	0.9500	O1S—H1SO	1.06 (4)
C9—C10	1.387 (2)	C1S—C2S	1.481 (3)
C9—C14	1.397 (2)	C1S—H1SA	0.9900
C10—C11	1.388 (2)	C1S—H1SB	0.9900
C10—H10A	0.9500	C2S—H2SA	0.9800
C11—C12	1.389 (2)	C2S—H2SB	0.9800
C11—H11A	0.9500	C2S—H2SC	0.9800
C12—C13	1.377 (2)	O1W—H1W	1.06 (3)
			
C8—O1—H1O	108.3 (15)	C17—C16—C21	121.72 (14)
C14—O2—H2O	108.4 (14)	C17—C16—C15	130.12 (14)
C15—N1—C22	111.07 (11)	C21—C16—C15	108.13 (12)
C15—N1—C1	120.88 (11)	C16—C17—C18	117.14 (16)
C22—N1—C1	128.01 (11)	C16—C17—H17A	121.4
C23—N2—C30	111.23 (11)	C18—C17—H17A	121.4
C23—N2—C2	125.82 (11)	C19—C18—C17	121.30 (16)
C30—N2—C2	122.64 (11)	C19—C18—H18A	119.4
N1—C1—C3	111.94 (11)	C17—C18—H18A	119.4
N1—C1—C2	109.82 (11)	C18—C19—C20	121.61 (15)
C3—C1—C2	114.75 (11)	C18—C19—H19A	119.2
N1—C1—H1A	106.6	C20—C19—H19A	119.2
C3—C1—H1A	106.6	C21—C20—C19	116.56 (15)
C2—C1—H1A	106.6	C21—C20—H20A	121.7
N2—C2—C9	110.91 (10)	C19—C20—H20A	121.7
N2—C2—C1	108.73 (11)	C16—C21—C20	121.66 (14)
C9—C2—C1	114.44 (11)	C16—C21—C22	108.45 (13)
N2—C2—H2A	107.5	C20—C21—C22	129.89 (14)
C9—C2—H2A	107.5	O4—C22—N1	126.39 (12)
C1—C2—H2A	107.5	O4—C22—C21	127.65 (13)
C4—C3—C8	118.56 (13)	N1—C22—C21	105.96 (12)
C4—C3—C1	122.80 (13)	O5—C23—N2	125.38 (13)
C8—C3—C1	118.64 (13)	O5—C23—C24	128.16 (13)
C5—C4—C3	121.22 (15)	N2—C23—C24	106.45 (11)
C5—C4—H4A	119.4	C25—C24—C29	121.47 (14)
C3—C4—H4A	119.4	C25—C24—C23	130.33 (13)
C6—C5—C4	119.56 (15)	C29—C24—C23	108.16 (12)
C6—C5—H5A	120.2	C24—C25—C26	117.33 (14)
C4—C5—H5A	120.2	C24—C25—H25A	121.3
C5—C6—C7	120.41 (14)	C26—C25—H25A	121.3
C5—C6—H6A	119.8	C27—C26—C25	121.36 (14)
C7—C6—H6A	119.8	C27—C26—H26A	119.3
C6—C7—C8	120.02 (16)	C25—C26—H26A	119.3
C6—C7—H7A	120.0	C26—C27—C28	120.89 (14)
C8—C7—H7A	120.0	C26—C27—H27A	119.6
O1—C8—C7	121.83 (14)	C28—C27—H27A	119.6
O1—C8—C3	117.96 (13)	C29—C28—C27	117.36 (14)
C7—C8—C3	120.21 (15)	C29—C28—H28A	121.3
C10—C9—C14	118.27 (13)	C27—C28—H28A	121.3
C10—C9—C2	123.96 (12)	C28—C29—C24	121.58 (14)
C14—C9—C2	117.72 (12)	C28—C29—C30	130.44 (13)
C9—C10—C11	121.31 (13)	C24—C29—C30	107.96 (12)
C9—C10—H10A	119.3	O6—C30—N2	124.71 (13)
C11—C10—H10A	119.3	O6—C30—C29	129.13 (13)
C10—C11—C12	119.53 (14)	N2—C30—C29	106.16 (12)
C10—C11—H11A	120.2	C1S—O1S—H1SO	112.9 (17)
C12—C11—H11A	120.2	O1S—C1S—C2S	108.83 (16)
C13—C12—C11	120.16 (14)	O1S—C1S—H1SA	109.9
C13—C12—H12A	119.9	C2S—C1S—H1SA	109.9
C11—C12—H12A	119.9	O1S—C1S—H1SB	109.9
C12—C13—C14	120.01 (14)	C2S—C1S—H1SB	109.9
C12—C13—H13A	120.0	H1SA—C1S—H1SB	108.3
C14—C13—H13A	120.0	C1S—C2S—H2SA	109.5
O2—C14—C13	122.18 (13)	C1S—C2S—H2SB	109.5
O2—C14—C9	117.10 (13)	H2SA—C2S—H2SB	109.5
C13—C14—C9	120.71 (14)	C1S—C2S—H2SC	109.5
O3—C15—N1	124.43 (14)	H2SA—C2S—H2SC	109.5
O3—C15—C16	129.23 (13)	H2SB—C2S—H2SC	109.5
N1—C15—C16	106.32 (11)		
			
C15—N1—C1—C3	116.36 (13)	O3—C15—C16—C21	175.63 (15)
C22—N1—C1—C3	−65.99 (17)	N1—C15—C16—C21	−2.64 (15)
C15—N1—C1—C2	−114.96 (13)	C21—C16—C17—C18	−0.9 (2)
C22—N1—C1—C2	62.69 (17)	C15—C16—C17—C18	177.31 (15)
C23—N2—C2—C9	−55.50 (17)	C16—C17—C18—C19	0.7 (3)
C30—N2—C2—C9	131.43 (13)	C17—C18—C19—C20	0.0 (3)
C23—N2—C2—C1	71.16 (16)	C18—C19—C20—C21	−0.5 (2)
C30—N2—C2—C1	−101.91 (14)	C17—C16—C21—C20	0.4 (2)
N1—C1—C2—N2	51.45 (13)	C15—C16—C21—C20	−178.14 (12)
C3—C1—C2—N2	178.57 (11)	C17—C16—C21—C22	−179.33 (14)
N1—C1—C2—C9	176.06 (10)	C15—C16—C21—C22	2.09 (15)
C3—C1—C2—C9	−56.82 (15)	C19—C20—C21—C16	0.3 (2)
N1—C1—C3—C4	73.68 (16)	C19—C20—C21—C22	180.00 (15)
C2—C1—C3—C4	−52.35 (18)	C15—N1—C22—O4	178.83 (13)
N1—C1—C3—C8	−105.50 (14)	C1—N1—C22—O4	1.0 (2)
C2—C1—C3—C8	128.47 (14)	C15—N1—C22—C21	−0.94 (15)
C8—C3—C4—C5	1.6 (2)	C1—N1—C22—C21	−178.78 (12)
C1—C3—C4—C5	−177.58 (13)	C16—C21—C22—O4	179.46 (14)
C3—C4—C5—C6	−1.7 (2)	C20—C21—C22—O4	−0.3 (2)
C4—C5—C6—C7	0.6 (2)	C16—C21—C22—N1	−0.78 (15)
C5—C6—C7—C8	0.6 (2)	C20—C21—C22—N1	179.48 (14)
C6—C7—C8—O1	179.03 (15)	C30—N2—C23—O5	176.69 (13)
C6—C7—C8—C3	−0.7 (2)	C2—N2—C23—O5	3.0 (2)
C4—C3—C8—O1	179.85 (13)	C30—N2—C23—C24	−1.99 (15)
C1—C3—C8—O1	−0.9 (2)	C2—N2—C23—C24	−175.74 (11)
C4—C3—C8—C7	−0.4 (2)	O5—C23—C24—C25	0.8 (2)
C1—C3—C8—C7	178.83 (14)	N2—C23—C24—C25	179.41 (14)
N2—C2—C9—C10	99.23 (15)	O5—C23—C24—C29	−177.04 (13)
C1—C2—C9—C10	−24.22 (18)	N2—C23—C24—C29	1.60 (15)
N2—C2—C9—C14	−78.18 (15)	C29—C24—C25—C26	1.1 (2)
C1—C2—C9—C14	158.37 (13)	C23—C24—C25—C26	−176.41 (14)
C14—C9—C10—C11	−0.6 (2)	C24—C25—C26—C27	−0.9 (2)
C2—C9—C10—C11	−177.99 (13)	C25—C26—C27—C28	−0.3 (2)
C9—C10—C11—C12	−0.3 (2)	C26—C27—C28—C29	1.2 (2)
C10—C11—C12—C13	0.4 (2)	C27—C28—C29—C24	−1.0 (2)
C11—C12—C13—C14	0.5 (2)	C27—C28—C29—C30	177.11 (14)
C12—C13—C14—O2	177.19 (14)	C25—C24—C29—C28	−0.2 (2)
C12—C13—C14—C9	−1.4 (2)	C23—C24—C29—C28	177.82 (13)
C10—C9—C14—O2	−177.22 (13)	C25—C24—C29—C30	−178.67 (13)
C2—C9—C14—O2	0.34 (19)	C23—C24—C29—C30	−0.63 (15)
C10—C9—C14—C13	1.5 (2)	C23—N2—C30—O6	−177.39 (13)
C2—C9—C14—C13	179.05 (13)	C2—N2—C30—O6	−3.4 (2)
C22—N1—C15—O3	−176.19 (14)	C23—N2—C30—C29	1.62 (15)
C1—N1—C15—O3	1.8 (2)	C2—N2—C30—C29	175.60 (11)
C22—N1—C15—C16	2.18 (15)	C28—C29—C30—O6	0.1 (3)
C1—N1—C15—C16	−179.80 (11)	C24—C29—C30—O6	178.40 (14)
O3—C15—C16—C17	−2.8 (3)	C28—C29—C30—N2	−178.82 (14)
N1—C15—C16—C17	178.95 (15)	C24—C29—C30—N2	−0.55 (15)

### Hydrogen-bond geometry (Å, º)

**Table:**

*D*—H···*A*	*D*—H	H···*A*	*D*···*A*	*D*—H···*A*
O1—H1*O*···O1*W*	0.86 (2)	1.89 (2)	2.7263 (13)	165 (2)
O2—H2*O*···O5^i^	0.88 (2)	1.95 (2)	2.8238 (15)	177 (2)
O1*S*—H1*SO*···O4	1.06 (4)	1.76 (4)	2.7905 (17)	161 (3)
O1*W*—H1*W*···O1*S*^ii^	1.06 (3)	1.65 (3)	2.6927 (16)	169 (3)

Symmetry codes: (i) −*x*−1/2, *y*−1/2, −*z*+1/4; (ii) *x*, *y*+1, *z*.
